# Alcohol Relapse After Liver Transplantation: Advances in Risk Stratification, Biomarker Integration, and Post-Transplant Care

**DOI:** 10.1016/j.gastha.2025.100853

**Published:** 2025-12-02

**Authors:** Vincent Pedulla, Alyson Kaplan

**Affiliations:** 1 Tufts University School of Medicine, Boston, Massachusetts; 2 Abdominal Transplant Institute, Tufts Medical Center, Boston, Massachusetts

**Keywords:** Liver Transplantation, Alcohol Use Disorder, Relapse Prediction, Phosphatidylethanol, Social Determinants of Health, Transplant Selection

## Abstract

Alcohol-associated liver disease (ALD) is now the primary indication for liver transplantation (LT) in the United States. While outcomes after LT for ALD are generally excellent, the possibility of post-LT alcohol relapse raises ongoing clinical, ethical, and psychosocial challenges. Relapse is shaped by multiple factors, including young age, comorbid substance use or psychiatric history, lack of social support or engagement, and broader social determinants of health such as education, race, socioeconomic status, and geography. These influences are often difficult to capture through traditional psychosocial assessment alone, and program-level variation in evaluation practices may exacerbate disparities in access to LT. Several relapse prediction tools, including the Sustained Alcohol Use Post-Liver Transplant and Stanford Integrated Psychosocial Assessment for Transplant, have been developed to aid in candidate evaluation. While these tools provide structured approaches, their predictive accuracy remains limited. Biomarkers of alcohol use have emerged as valuable adjuncts to the psychosocial assessment, with phosphatidylethanol showing the greatest promise due to its high sensitivity and specificity and ability to detect alcohol use over a longer period of time. Post-transplant multidisciplinary treatment of alcohol use disorder is also important, including pharmacotherapy and addiction care. Ultimately, optimizing relapse prediction and management requires a framework that accounts not only for individual risk factors but also for structural inequities that shape access to transplantation. Efforts to combine clinical, biological, and social data into unified risk models may provide a more equitable and evidence-based approach to evaluating and supporting patients with ALD before and after LT.

## Introduction

Liver failure secondary to alcohol use disorder (AUD) remains a major cause of morbidity and mortality worldwide.^1^^,^^2^ Since about 2015, alcohol-associated liver disease (ALD) has become the leading indication for liver transplantation (LT), surpassing hepatitis C following the advent of effective antiviral therapy.^3^ Unlike patients with kidney failure, who can often be bridged to transplant with dialysis, there is no equivalent life-sustaining therapy for patients with end-stage liver disease.^4^ LT is the only definitive treatment; without it, patients can face severe complications including ascites, spontaneous bacterial peritonitis, hepatic encephalopathy, hepatorenal syndrome, and hepatocellular carcinoma.^5^ Unfortunately, the demand for donor organs far exceeds supply, and many patients either do not qualify for transplant or die while waiting. This reality underscores the critical need to identify candidates who are most likely to succeed after transplant, particularly regarding the risk of alcohol relapse. Yet there is no single validated tool that reliably predicts relapse. Rates of return to alcohol use after LT remain high, with up to 30% relapsing to harmful drinking and over one-third of those individuals developing recurrent cirrhosis.^6^ Return to harmful drinking is associated with worse outcomes including graft rejection, steatohepatitis, cardiovascular disease, and death.^7^, ^8^, ^9^, ^10^

Historically, the debate around transplantation for ALD has centered on pretransplant abstinence requirements, with the “6-month rule” dominating discussions for decades.^4^^,^^11^ However, growing evidence has demonstrated that fixed abstinence intervals are poor predictors of long-term sobriety and may unfairly exclude patients with high medical urgency.^11^ As a result, attention has shifted toward identifying reliable predictors of relapse, developing validated risk stratification tools, and implementing strategies to support sustained abstinence after LT.

Multiple domains influence relapse risk, including psychiatric comorbidity, social support, and co-use of other substances. Psychosocial scoring systems such as the Sustained Alcohol Use Post-Liver Transplant (SALT) score, the Stanford Integrated Psychosocial Assessment for Transplant (SIPAT), and the High-Risk Alcoholism Relapse (HRAR) scale have been evaluated, though their predictive performance remains imperfect. In parallel, biochemical markers of alcohol use—particularly phosphatidylethanol (PEth)—have emerged as objective tools to complement psychosocial assessments. These approaches underscore the need for multimodal evaluation frameworks that integrate behavioral, social, and biologic factors.

Equally important is the recognition that relapse risk does not end at the time of transplantation. Evidence increasingly highlights the value of structured post-LT interventions, including addiction treatment, pharmacotherapy for AUD, and multidisciplinary follow-up models designed to promote long-term recovery. Furthermore, equity concerns, including socioeconomic disparities, geographic barriers, and uneven access to transplant centers, continue to influence which patients can receive LT for ALD.

In this review, we summarize current knowledge on predictors of alcohol relapse, both before and after LT, examine the role of psychosocial scoring systems and biomarkers, and highlight emerging models of post-transplant care. We also explore broader considerations of access and equity in LT for ALD, aiming to provide a framework for future practice and research.

## Risk Factors for Relapse Post-Liver Transplantation

While numerous clinical, behavioral, and social factors have been evaluated as predictors of alcohol relapse following liver transplantation (LT), no single variable offers perfect discrimination, and data often vary between cohorts. Increasingly, studies are moving beyond binary abstinence requirements toward more nuanced approaches that consider risk factors within a broader psychosocial and clinical context. This section summarizes key factors associated with post-LT relapse, including age, comorbid substance use, prior treatment history, abstinence duration, nonadherence, psychiatric comorbidity, social support, and employment.

### Age

Younger age is one of the most consistently reported risk factors for post-LT alcohol relapse.^12^^,^^13^ Several studies have shown a linear relationship between increasing age and reduced relapse risk, with a 4% decrease in relapse hazard for each additional year of age.^9^^,^^14^^,^^15^ Patients under 40 years old appear to be at particularly high risk.^16^ Younger individuals may face unique challenges, including limited time to engage in AUD treatment prior to transplant, social pressures, and a greater likelihood of denial or minimization of disease severity.^17^ Additionally, discordance between self-reported abstinence and PEth positivity is more common in younger patients, suggesting that this population may benefit from enhanced screening and monitoring.^18^

### Comorbid Substance Use

The presence of comorbid substance use (beyond alcohol) raises concern among transplant clinicians, both due to associated health risks in the post-transplant period and the potential indication of broader difficulties with behavioral control and abstinence. Tobacco use has been the most consistently identified comorbid substance associated with increased risk of post-LT alcohol relapse.^13^^,^^14^^,^^19^^,^^20^ Evidence also suggests that polysubstance use—defined as the use of multiple substances—is a predictor of relapse, including both any alcohol use and heavy relapse.^20^ While individual substances such as cannabis and heroin have not been consistently associated with increased relapse risk, cocaine use, in particular, has been linked to heavy post-LT relapse.^13^ These findings build on prior work demonstrating that comorbid substance use may accelerate time to first drink following LT.^21^ Ongoing investigation is needed to clarify which substances and what consumption patterns most strongly predict relapse risk and how these should be incorporated into structured risk assessment.

### Prior Relapse, Abstinence Duration, and Nonadherence

A history of failed sobriety attempts or prior relapse significantly increases the risk of post-LT harmful drinking.^12^^,^^22^ One study found a 199% increase in the odds of harmful relapse among those with a prior history of relapse.^23^ However, the utility of this information depends heavily on the context, including the frequency, severity, and recency of relapse, as well as the patient’s response to prior treatment efforts. Historically, a “6-month rule” of sobriety prior to LT was widely enforced, under the assumption that this period served as a test to see if patients recovered as well as a test of behavioral change and predictor of post-LT abstinence. While early data supported this practice, these studies often failed to account for modern treatment approaches, evolving population demographics, or nuanced definitions of relapse.^16^^,^^24^

More recent data have challenged the validity and ethics of rigid sobriety requirements. For example, multicenter studies have found no clear association between the duration of pre-LT sobriety and the risk of post-LT relapse, and some suggest that survival outcomes are comparable—or even improved—in patients who undergo early transplant without fulfilling the traditional 6-month benchmark.^9^^,^^11^^,^^25^^,^^26^ When associations between length of sobriety and rates of relapse have been described, they demonstrated a linear relationship between abstinence duration and reduced relapse risk, but not a particular threshold of time that would be required. For instance, each additional month of sobriety has been associated with a 4%–33% reduction in relapse odds, though there has not been a specific identified length of sobriety after which risk falls significantly.^15^^,^^21^^,^^23^

These findings have translated into a marked shift in clinical practice. In one national survey, only 21% of US transplant centers now enforce a strict 6-month sobriety rule, and many others use shorter time frames or assess readiness based on individualized criteria.^27^ A major driver of this shift has been the growing acceptance of liver transplant for alcohol-associated hepatitis (AAH). Several studies have demonstrated excellent 1- and 5-year survival outcomes in patients undergoing early LT for AAH, even without prolonged abstinence.^27^, ^28^, ^29^, ^30^, ^31^ The Dallas Consensus Conference further reinforced that fixed sobriety periods should not be absolute requirements for transplant eligibility, particularly in patients with AAH who lack alternatives to curative treatment.^32^ Importantly, sobriety duration is not always a proxy for recovery or a predictor of relapse. For example, brief sobriety with active engagement in treatment may signal a high readiness for recovery. Thus, duration of abstinence should be considered alongside other factors, such as participation in treatment, insight into illness, social stability, and psychiatric comorbidities, rather than used as a binary cutoff.

Nonadherence to medical recommendations, such as continued alcohol use after ALD diagnosis, missed appointments, and failure to engage in an outpatient intensive treatment program, is also predictive of relapse. This behavior is often interpreted as a sign of limited insight or readiness for transplant and several studies support its use as a relative contraindication.^7^^,^^22^^,^^25^^,^^29^ Nevertheless, some have cautioned that nonadherence may be influenced by factors such as health literacy, mistrust in the medical system, or poor communication, and should be interpreted in context. For example, studies rarely assess whether patients were adequately educated about their disease, and low educational attainment itself has been linked to higher relapse risk.^15^

### Psychiatric Comorbidity

Psychiatric illness is a well-documented predictor of post-LT relapse. Conditions such as depression, anxiety, bipolar disorder, and schizophrenia have all been associated with increased risk and frequently coexist with AUD, thereby contributing to both the development and persistence of harmful drinking behaviors.^9^ The relationship is likely bidirectional—psychiatric illness can lead to alcohol use as a form of self-medication, and chronic alcohol consumption can exacerbate or trigger psychiatric symptoms.

Multiple studies have quantified the impact of psychiatric diagnoses on post-LT outcomes. One study found a hazard ratio of 2.83 for alcohol relapse following LT among patients with any psychiatric comorbidity, and another reported an odds ratio of 5.22 in post-LT recipients with at least one diagnosed psychiatric condition, even when medically controlled.^9^^,^^25^ Moreover, even well-controlled psychiatric conditions were associated with higher rates of harmful drinking post-LT, underscoring that psychiatric history, rather than active symptom burden alone, can influence relapse risk.^19^

Importantly, the diagnosis of a psychiatric condition should not automatically exclude a patient from transplant eligibility. While patients with unstable or high-risk psychiatric profiles, such as active suicidality or psychosis, are generally excluded, milder conditions remain common and warrant integrated pre- and post-transplant mental health support.

### Social Support

Social support plays a critical role in maintaining post-LT abstinence. Most transplant centers consider the absence of a reliable support system a relative contraindication to listing, particularly for patients with ALD.^29^ While logistical needs such as transportation to appointments and caregiving are important, emotional and accountability support are equally vital in preventing relapse. Interestingly, the presence of a partner appears protective only under certain conditions—patients who were recently widowed or divorced showed increased relapse risk, while those who had been single for a long time pre-LT did not.^9^^,^^25^ Additionally, having young children in the home has been linked to higher relapse rates compared to having adult children, though the reasons for this remain unclear.^16^ Lastly, the quality of support also matters. For example, living with someone who drinks or does not support abstinence may be more detrimental than living alone.

### Employment and Social Engagement

Although social integration, including employment, has been hypothesized to reduce relapse risk, the data are mixed. ALD patients are less likely to be employed at the time of transplant than those with other liver disease etiologies; however, employment itself has not been consistently shown to be protective.^29^ In fact, studies using PEth-based assessments found no significant association between employment status and relapse risk.^9^ Financial instability may be a better predictor of relapse than employment; almost half of post-LT ALD patients who relapsed reported financial stressors at the time of transplant as compared to only 29% in the group that did not relapse.^22^ These findings suggest that while employment and financial security may contribute to broader markers of social stability, neither appears to independently predict post-LT alcohol use.

### Summary

The prediction of alcohol relapse after LT remains imperfect, though several factors consistently emerge as high-yield risk indicators. Still, no single variable should be used in isolation to decline a patient for transplant. For example, disqualifying a patient based solely on age would be both unethical and unsupported by evidence. Instead, these risk factors should be considered together when making decisions about transplant candidacy. Additionally, they can help to inform individualized care plans, identifying patients who may benefit from enhanced monitoring, counseling, or structured support. Ongoing research and a shift toward equitable, multidimensional assessment are critical to improving access to transplant for patients with AUD.

## Scoring Systems

While identifying individual predictors of relapse is an important first step, transplant centers often need structured, reproducible tools to assess risk in a consistent and equitable way. To that end, prognostic scoring systems have been developed to synthesize various clinical, psychosocial, and behavioral risk factors into a quantifiable framework. However, determining which tools offer the greatest predictive validity and clinical utility remains an ongoing challenge. While several models have been commonly used, few have been prospectively validated in diverse patient populations, and guidance on how to interpret and apply individual scores in real-world transplant decision-making is limited. Establishing the most reliable tools and standardizing their use is a key area of ongoing research in the field. Table 1 includes the main prognostic screening tools used along with their benefits, drawbacks, sensitivity, specificity, positive predictive value (PPV), and negative predictive value (NPV).

**Table 1:** Characteristics of Screening Tools to Predict Relapse Post-LT^2^^,^^3^^,^^6^^,^^8^^,^^22^^,^^25^^,^^31^, ^32^, ^33^, ^34^, ^35^, ^36^, ^37^, ^38^, ^39^, ^40^, ^41^

Tool	Characteristics	Benefits	Drawbacks
AUDIT	10 items, developed by the WHO Sensitivity: 64%–86%^8^^,^^35^ Specificity: 74%–94%^8^^,^^35^ PPV: 72%–77%^40^ NPV: 80%^41^	Widely validated to correlate to EtOH consumption^6^^,^^33^^,^^34^ AUDIT-C allows for targeted assessment of recent alcohol use	Subjective screening, relies on self-reporting Significant discordance with PEth and other biomarker results^2^^,^^37^
AUDIT-C	3-item condensed version of the AUDIT Sensitivity: 80%^8^ Specificity: 70%–80%^8^ PPV: 24%–82%^36^ NPV: 87%–99%^36^	Shorter administration than the AUDIT	Lack of widespread evidence in the setting of LT
HRAR	Early test, 3 domains, each scored 0–2 (total range 0–6) Sensitivity: 37%–38%^31^ Specificity: 67%^31^ PPV: 22%^31^ NPV: 95%^31^	Multifactorial; assesses duration, average consumption, and rehabilitation attempts Score of 3–4 validated as a threshold for high risk of post-LT relapse^25^^,^^31^ Favorable NPV	Low PPV Does not account for comorbid psychiatric conditions, social support, or co-occurring substance use
SALT	4 domains, score range 0–11 Sensitivity: 37%–47%^31^ Specificity: 66%–68%^31^ PPV: 26%^31^ NPV: 83%^31^	Multifactorial; assesses consumption, rehabilitation attempts, past drug use, and EtOH-related legal issues, derived from multicenter data^38^ Favorable NPV	Developed using early-LT patients with AAH; might not apply to chronic ALD Low PPV Lack of agreed upon threshold score^22^
SIPAT	18 items, range of 0–119, ordinal grouping (excellent, good, minimally acceptable, poor, or high risk) Sensitivity: 78%^25^ Specificity: 51%^25^ PPV: 16%^25^ NPV: 95%^25^	Widely used among LT centers Multidisciplinary; assesses readiness, social support, psychologic suitability, psychopathology, and substance use^39^	Not specific to ALD Takes longer to administer than other tools

Sensitivity, specificity, PPV, and NPV data may depend on the primary study’s cutoff threshold for each test.

LT, liver transplantation; WHO, World Health Organization.

### Alcohol Use Disorders Identification Test

The Alcohol Use Disorders Identification Test (AUDIT) is among the most widely used screening tools for AUD. The 10-item survey, developed by the World Health Organization, can detect patterns of AUD, as high scores have been shown to correlate with alcohol-related hospitalizations.^33^^,^^34^ The AUDIT-Consumption (AUDIT-C) is a shortened, 3-item version focused on recent drinking behavior.^35^ AUDIT’s sensitivity is estimated to range from 64% to 86%, potentially limited by underreporting due to stigma, fear of transplant denial, and cognitive impairment from hepatic encephalopathy.^8^ Moreover, concerns have been raised about its predictive validity. For example, in a Portuguese study of patients 2 years post-transplant, all participants who self-reported a return to alcohol consumption scored “low risk” on the AUDIT.^14^ Therefore, in this population, AUDIT underestimated relapse risk and misclassified patients. The AUDIT-C’s sensitivity and specificity are similar to those of the AUDIT, with much stronger NPVs for all cutoff scores than PPV (87%–99% versus 24%–82%, respectively.^36^ These findings, however, are not validated widely, if at all, in the setting of post-LT relapse prediction.

Given the subjective nature of AUDIT-based assessments, increasing attention has turned to objective biomarkers such as PEth, which can directly detect recent alcohol use and is discussed in more detail below. Several studies have compared PEth results with AUDIT or AUDIT-C scores, often revealing significant discordance. For instance, in a study of healthy volunteers, PEth levels only aligned with AUDIT-C scores in those reporting total abstinence. Among participants who were classified as “excessive drinking,” some had undetectable PEth levels. Ninety-five percent of those classified as “moderate drinkers” actually had PEth concentrations well below the expected range.^37^ Similarly, a 2020 study of 50 patients with AUD or ALD found that 37.5% of those with a positive AUDIT score (8 or above) had negative results across PEth, ethyl-glucuronide (EtG), ethyl-sulfate (EtS), and hair ethyl glucuronide testing.^2^ These inconsistencies suggest that AUDIT-based screening alone may not be sufficient for screening.

### HRAR

The HRAR score is one of the earliest structured tools developed to estimate the likelihood of alcohol relapse following LT. It incorporates three factors that reflect the severity and chronicity of AUD: (1) the duration of heavy drinking, (2) the average number of daily drinks, and (3) the number of prior inpatient rehabilitation attempts. Each variable is scored from 0 to 2, yielding a total score that ranges from 0 to 6.^25^

A score of 4 or greater is commonly used as a threshold indicating high relapse risk, although some studies assume a threshold of 3 or greater.^25^^,^^31^ Despite its simplicity and historical use, the HRAR score has demonstrated limited predictive validity in contemporary post-LT populations. In one study of patients undergoing LT for AAH, HRAR did not independently predict sustained alcohol use post-transplant.^12^ Furthermore, in a direct comparison with PEth, HRAR demonstrated low sensitivity (37%) for detecting relapse, though its NPV was favorable, suggesting that a low score may be useful to help rule out risk.^31^

Some studies have found variable thresholds to be predictive in different populations. For example, one study found that an HRAR score ≥3 was predictive of relapse in patients without AAH, while another reported that a score ≥4 was more strongly associated with relapse risk.^13^^,^^23^ These discrepancies highlight the need for further validation and potential recalibration of the HRAR score in modern LT cohorts, particularly those that include patients with AAH, evolving listing practices, and those monitored with objective biomarkers like PEth.

### SALT

The SALT score was developed through retrospective analysis of individuals who underwent early LT for AAH, a setting in which minimal or no sobriety period was required.^12^ As such, the SALT score (with a range of 0–11) is primarily intended for use in assessing relapse risk among patients considered for early transplant without a period of prolonged abstinence.^38^

The original study from which the SALT score was derived identified a score of >5 as the threshold at which the risk of post-LT relapse increased, with a reported PPV of 25%.^12^ However, subsequent validation efforts have challenged the score’s predictive strength. One study found that only scores of 7 or higher were significantly associated with shorter time to relapse, calling into question the reliability of the originally proposed cutoff.^22^ In general, the SALT score has demonstrated low PPV but relatively favorable NPV, meaning it performs better in ruling out high-risk individuals than in confidently identifying them.^31^ This trend was further reflected in a recent study that found that the SALT score failed to identify several patients who ultimately relapsed to sustained alcohol use.^38^ Given these limitations, the SALT score should not be used in isolation to determine transplant eligibility. Rather, a high SALT score should prompt a more detailed psychosocial and behavioral assessment, while a low score may provide some reassurance of lower relapse risk.

### SIPAT

The SIPAT was developed in 2013 and is one of the most widely used structured tools for assessing psychosocial risk in transplant candidates. It evaluates four domains: patient readiness, social support, psychologic suitability and psychopathology, and substance use. While SIPAT is not specific to ALD, it captures many relevant dimensions of risk in this population, including psychiatric comorbidities (ie depression, anxiety), cognitive impairment (ie encephalopathy), coping skills, personality traits, and maladaptive behaviors such as deception and nonadherence.^25^

SIPAT scores range from 0 to 119 but can be grouped ordinally, categorizing patients as “excellent,” “good,” “minimally acceptable,” “poor,” or “high risk” transplant candidates.^25^ SIPAT’s utility in LT for ALD has only recently been studied.^39^ Recent data suggest that higher SIPAT scores are associated with increased risk of post-transplant relapse.^22^ For example, Sedki et al. (2024) identified a SIPAT score of >30 as a threshold above which relapse risk significantly increased.^25^ Deutsch-Link et al. (2020) similarly found that scores >21 were associated with lower likelihood of being rated “excellent” or “good candidates” and higher rates of post-LT relapse.^22^ However, a significant proportion of patients who remain abstinent post-LT had SIPAT scores above the previously described thresholds, underscoring that elevated psychosocial risk does not necessarily equate to higher relapse rates.^22^^,^^25^ In fact, a retrospective multicenter study found no association between SIPAT score and post-transplant alcohol use as measured by PEth testing.^9^ This discrepancy highlights the complexity of predicting relapse and the need to interpret SIPAT scores within the broader clinical and behavioral context.

Compared to other tools, SIPAT appears to offer broader utility. It is often favored over the SALT score and the HRAR score.^25^ Still, SIPAT is not without limitations. One commonly cited critique is its subjective scoring, which can vary depending on the evaluator’s interpretation and clinical training.^31^ Standardization of the administration and inter-rater reliability remain areas for improvement.

### Summary

In summary, prognostic scoring systems such as AUDIT, SALT, and SIPAT offer valuable frameworks for assessing relapse risk in LT candidates with ALD. Each tool has unique strengths—AUDIT for screening harmful alcohol use, SALT for considerations of early LT without long periods of sobriety, and SIPAT for comprehensive psychosocial evaluations—but all are limited by issues of subjectivity, underreporting, or insufficient predictive value when used in isolation. Therefore, no single score should serve as the sole determinant of transplant eligibility. Instead, they should inform a more holistic assessment that incorporates objective biomarkers, clinical judgment, and multidisciplinary input. Future work should focus on refining these models, validating them across diverse cohorts, and integrating them into standardized transplant evaluation pathways to ensure equitable and evidence-based candidate selection.

## Biomarkers

Screening for alcohol (EtOH) use with biomarkers has long been a topic of interest due to the potential for objective evaluation in the LT selection process. Table 2 shows the detection windows for major biomarkers used in LT evaluation.

**Table 2:** Biomarker EtOH Detection^42^, ^43^, ^44^

Test	Detection window	Sensitivity, specificity (%)	PPV (%)	NPV (%)
PEth	28 d	90, 100^45^	85^43^	100^43^
EtG	5 d	60, 95^45^		
u-EtG/u-EtS	3–5 d	71, 98^43^	89^44^	99^44^
h-EtG	5–6 mo^45^	84, 92^43^	68^43^	96^43^
Ethanol/methanol	2 d	>90^45^	75^44^	93^44^
CDT	2–4 wk	25–85, 70–98^45^	100^43^	86^43^

CDT, carbohydrate deficient transferrin; h-EtG, hair ethyl glucuronide; LT, liver transplantation; u-EtG, urine ethyl glucuronide; u-EtS, urine ethyl sulfate.

### Traditional Biomarkers

Traditional markers of alcohol use include indirect biomarkers (physiologic or biochemical changes associated with heavy alcohol use) as well as direct metabolites of alcohol. Indirect markers, such as gamma-glutamyltransferase (GGT), mean corpuscular volume (MCV), and carbohydrate-deficient transferrin have been studied extensively. GGT and MCV are inexpensive and widely available but lack specificity, as both can be elevated due to liver disease, medications, or nutritional deficiencies, factors that are common in patients with ALD.^46^ Carbohydrate-deficient transferrin performs well in detecting alcohol intake but does not allow for the distinction between low and moderate intake.^47^

Direct metabolites provide stronger evidence of recent alcohol intake. Among these, urine ethyl glucuronide and EtS are widely used. Urinary markers often have shorter detection windows (3–5 days), making them useful for identifying very recent drinking. However, they are susceptible to manipulation (ie dilution, substitution) and may be confounded by liver or kidney dysfunction.^2^^,^^7^^,^^42^^,^^48^^,^^49^ In contrast, hair EtG offers a longer detection window of up to several months; however, it is limited by variability in hair length and growth, cosmetic treatments, and lack of standardized transplant-specific cutoffs.^43^^,^^44^

### PEth: A Contemporary Biomarker

PEth has emerged as the most sensitive and specific biomarker for detecting recent alcohol use in liver transplantation (LT) candidates.^42^^,^^50^^,^^51^ PEth is a direct alcohol biomarker formed only in the presence of ethanol via a reaction between ethanol and phosphatidylcholine in red blood cell membranes.^10^ Unlike other markers, PEth is not enzymatically degraded and has a half-life of approximately 28 days, allowing detection of alcohol intake over several weeks.^43^^,^^52^^,^^53^ Its formation requires ethanol, making false positives extremely rare. Importantly, PEth is detectable through routine blood draws and quantifiable: levels above 20 ng/mL typically indicate recent alcohol use, with thresholds above 250–400 ng/mL suggesting moderate to heavy consumption.^34^^,^^37^ This dynamic range makes PEth a valuable adjunct to self-report and other screening tools, particularly in populations where underreporting is common. For example, a subset of patients with varying liver disease and AUD status tested positive for PEth despite reports of complete abstinence.^54^ Because of its reliability, PEth is now used by over 90% of U.S. transplant centers.^9^^,^^27^

PEth offers distinct advantages over other biomarkers. PEth has demonstrated superior sensitivity in post-LT populations, detecting alcohol use in patients missed by EtG testing in up to half of cases.^7^^,^^45^ PEth, used in conjunction with urine ethyl glucuronide, was able to detect 75% of recent alcohol use.^43^ Therefore, pairing PEth with other tests may increase sensitivity, especially when used in high-stakes decision-making such as transplant listing.

The reclassification of steatotic liver disease (SLD) into metabolic dysfunction and alcohol-associated liver disease (MetALD) and metabolic dysfunction-associated steatotic liver disease (MASLD) has important implications for the transplant evaluation and role of alcohol biomarkers. MASLD is characterized by hepatic steatosis in the presence of metabolic risk factors and minimal or no alcohol intake, whereas MetALD refers to individuals who meet metabolic criteria but consume alcohol above low-risk thresholds, creating an overlapping phenotype. ALD remains the primary diagnosis for patients with significant alcohol use and alcohol-associated liver injury.^55^

Emerging evidence suggests that alcohol use patterns and relapse risk may differ across these groups. PEth has demonstrated particular value in differentiating MASLD from MetALD and ALD in pretransplant settings, allowing clinicians to more accurately characterize alcohol exposure and tailor psychosocial assessment and intervention. For example, in a sample of SLD patients, PEth largely outperformed traditional biomarkers such as alanine aminotransferase, aspartate aminotransferase, MCV, GGT, and the ALD/NAFLD index in the detection of ALD.^56^ As metabolic and alcohol-related liver disease increasingly coexist, incorporating sensitive biomarkers such as PEth into standardized evaluation protocols will be essential to ensure consistency, equity, and accuracy in transplant decision-making. PEth can therefore be a helpful tool in detecting alcohol use in patients with SLD.

In the context of LT, PEth has emerged as a promising tool for assessing both pre- and post-transplant alcohol use. In pretransplant evaluations, PEth can help identify patients with recent alcohol consumption who might not disclose it, thereby prompting further psychosocial assessment and targeted intervention. Post-transplant, PEth offers a noninvasive means of monitoring for alcohol relapse, including early or low-level drinking that may precede more significant relapse events. For example, PEth has identified significantly higher rates of alcohol use in post-LT patients than was disclosed through clinical interview or other testing.^52^ Similar findings have been reported in cohorts of patients with AAH undergoing early LT, a group often at heightened risk for relapse due to abbreviated sobriety periods.^57^ Additionally, PEth levels measured serially over time may help distinguish between single-use “slips” and patterns of recurrent use, aiding clinical decision-making about the need for addiction treatment escalation or enhanced monitoring.

Despite its strengths, PEth is not without limitations. Interpretation thresholds vary, especially when distinguishing moderate from heavy use. Some experts propose a conservative cutoff of 20 ng/mL for “any use,” while others suggest values above 250–500 ng/mL as indicative of sustained or heavy drinking.^34^ Additionally, PEth’s high specificity reduces the likelihood of false positives, but rare confounders do exist—most notably, recent blood transfusions within 28 days can produce spurious results due to circulating donor PEth.^43^^,^^51^^,^^58^ However, medications, comorbidities, and even auto-brewery syndrome have not been found to yield false positives.^58^ Additionally, hand sanitizer or food products containing trace alcohol have not been shown to produce positive PEth results.^59^ False negatives are possible, especially in the setting of very low or sporadic alcohol intake. In one Spanish study of patients with ALD and AUD, EtS was the most frequently positive biomarker, occasionally identifying use missed by PEth.^2^ These instances raise questions about whether PEth is sufficiently sensitive to detect trace drinking and whether that level of detection is clinically meaningful.

As PEth becomes more widely adopted, it also raises ethical and practical considerations. Positive results can lead to transplant denial, even in patients with otherwise appropriate candidacy, and there is concern that over-reliance on a single biomarker could lead to inequitable outcomes.^60^ Discordance between PEth results and patient self-reporting is not uncommon and may be explained by fear of stigmatization or disqualification. Younger age, in particular, has been associated with underreporting.^7^ Additionally, studies have shown high rates of positive PEth among patients without documented AUD, including those with non-ALD liver disease, when routine screening was expanded.^10^^,^^42^ This finding suggests the need for standardized PEth use across all transplant candidates to avoid bias and improve equity in selection.

Going forward, PEth is likely to play an increasing role in transplant protocols, not as a stand-alone gatekeeper, but as part of a comprehensive, patient-centered framework. Best practices may include using PEth alongside structured interviews (eg AUDIT-C, SIPAT, SALT, etc.), collateral histories, and social work and psychiatry input. While a positive PEth should not automatically disqualify a candidate, it can prompt deeper evaluation of readiness for transplant, the adequacy of relapse prevention strategies, and the need for ongoing support. Future research should prioritize standardization of PEth testing, define clinically meaningful thresholds, and explore how best to integrate biomarkers into shared decision-making processes in determining transplant candidacy.

## Post-LT Interventions to Prevent Relapse

Historically, much of the literature and clinical focus in ALD has centered on identifying pretransplant predictors of relapse, often with the goal of selecting appropriate candidates for LT. However, there has been comparatively less emphasis on structured, evidence-based interventions aimed at supporting sustained sobriety post-LT. As the field shifts toward a more holistic, longitudinal view of transplant success, including graft function, patient survival, and long-term quality of life, the importance of post-LT care has become increasingly clear. Transplant centers are now being evaluated not only on waitlist outcomes and perioperative metrics but also on their ability to improve quality of life in the post-transplant period. This section will examine strategies to reduce relapse risk post-LT, including ongoing addiction treatment, pharmacotherapy for AUD, and the critical role of coordinated, multidisciplinary follow-up.

### Addiction Treatment

Addiction treatment is a critical yet often underutilized component of post-LT care for patients with ALD. Despite the well-established relationship between AUD and LT, many transplant hepatologists report discomfort in managing relapse and a lack of formal training in addiction medicine.^27^ This gap in expertise, compounded by systemic barriers such as limited access to outpatient programs and long wait times, can undermine efforts to support long-term sobriety in the post-LT setting.

While addiction treatment has long been recognized as an important factor in promoting abstinence, it has only recently become a more prominent focus in the transplant literature. Early studies suggested that engagement in addiction treatment could significantly reduce post-LT relapse rates, yet for years, few programs integrated formal treatment into their post-LT care pathways.^61^ More recent data affirm the protective effect of structured substance use treatment that extends beyond the peritransplant period. In one study, patients who received both pre- and post-LT addiction treatment had significantly lower relapse rates (16%) than those who received no treatment (41%) or only pre-LT treatment (25%).^62^ It is clear that sustained engagement in therapy, relapse prevention planning, and behavioral health follow-up are essential, particularly in the high-risk months following LT when psychosocial stress, re-exposure to triggers, and declining external scrutiny may increase vulnerability to relapse.

### Pharmacologic Treatment

While psychosocial and behavioral interventions are crucial in maintaining abstinence after LT, pharmacologic treatment of AUD remains underutilized in transplant hepatology. Transplant providers often feel less comfortable prescribing medications for AUD, contributing to a lack of consistency in clinical practice and a relative dearth of transplant-specific data.^63^ The result is a persistent uncertainty regarding which pharmacologic regimens are most effective and safe in the post-LT setting. Additionally, the transplant population often presents with unique pharmacokinetic considerations—due to immunosuppression, renal function variation, and graft health—that complicate extrapolation from general AUD treatment studies.

The three medications most widely approved and used for AUD in the general population are disulfiram, naltrexone, and acamprosate. However, their use in patients with ALD and post-transplant status is complicated by concerns over safety and tolerability. Both naltrexone and disulfiram have been associated with potential hepatotoxicity, leading to hesitancy in using them in decompensated liver disease and after LT.^64^ Naltrexone, an opioid antagonist, has demonstrated anticraving properties and efficacy in preventing relapse in nontransplant AUD populations, but its hepatic metabolism and rare risk of liver injury limit have previously limited its use. Newer data have suggested that naltrexone can be safe, even in decompensated liver disease.^65^ Preliminary data show that injectable extended-release naltrexone, administered once a month, did not result in hepatotoxicity or liver decompensation for a monitoring period of over 4 months in advanced ALD patients.^66^ Therefore, it can likely be increasingly used in the post-LT setting as well, especially because it does not have any interactions with immunosuppressants.^67^

Because of concern over hepatotoxicity, attention has shifted toward medications with more favorable hepatic safety profiles, particularly acamprosate and baclofen.^5^ Acamprosate, which modulates glutamatergic neurotransmission and is primarily renally excreted, is generally considered safe in patients with liver dysfunction. However, until recently, data on its safety and efficacy in post-LT patients were lacking. A recent randomized controlled trial by Ayyala-Somayajula et al. (2025) was among the first to evaluate acamprosate specifically in liver transplant recipients.^68^ The study found that acamprosate was safe in this population and well tolerated, but it did not significantly reduce alcohol cravings or relapse compared to the standard of care, suggesting the need for further research to clarify its role in this context.

Baclofen, a gamma-aminobutyric acid-type B receptor agonist, has shown promise in patients with cirrhosis due to its ability to reduce alcohol cravings and anxiety and to promote abstinence. Early studies in patients with ALD, including those with cirrhosis, demonstrated both efficacy and safety.^69^^,^^70^ Baclofen is also predominantly renally cleared, making it an attractive option in patients with impaired liver function. However, despite its potential, there remains a lack of robust data on its efficacy in the post-transplant setting specifically. The absence of large, prospective studies in this population limits its widespread adoption.

Other medications occasionally considered in AUD treatment, such as topiramate, gabapentin, and varenicline, have been studied in nontransplant populations but have not been systematically evaluated for safety and efficacy after LT. Their off-label use may be considered on a case-by-case basis, particularly for patients with co-occurring psychiatric disorders or those who have not responded to first-line therapies.

Overall, there is a pressing need for more clinical research and guidance on pharmacologic interventions to support sobriety after liver transplantation. Despite growing recognition of their potential benefits, these medications remain underprescribed, often due to safety concerns, limited training, and lack of transplant-specific protocols. The integration of addiction specialists into transplant teams or increased education of transplant hepatologists can help bridge this gap and ensure that medication-assisted treatment is thoughtfully implemented as part of a comprehensive post-LT recovery plan.

### Multidisciplinary Care Post-LT

The post–LT period presents a critical window for relapse prevention, yet consistent and comprehensive care remains variable across centers. While many transplant programs conduct routine follow-up for medical management, structured protocols that explicitly address psychosocial well-being and relapse prevention are less uniformly applied. Survey data indicate that protocolized psychosocial follow-up is more common pretransplant than post-transplant, despite the ongoing risk of relapse during recovery.^27^ For example, in a national study of post-LT patient experiences, many individuals reported that alcohol use was rarely discussed in routine clinic visits, highlighting a missed opportunity for early identification and intervention.^23^ Additionally, a recent survey distributed to providers of multiple national organizations found that >50% of centers had no policy for prescribing pharmacotherapy for AUD, and >25% indicated a desire for increased interface between the patient and transplant center, including multidisciplinary care.^71^

Effective relapse prevention requires a multidisciplinary approach that goes beyond routine hepatology visits.^72^ Psychosocial stressors, psychiatric comorbidities, and social determinants of health can emerge or evolve post-LT and may not be readily apparent without intentional and ongoing screening. As such, transplant centers are increasingly recognizing the value of integrating addiction medicine, psychiatry, social work, and peer support into longitudinal care. There is evidence suggesting that cognitive behavioral therapy should have a role in relapse prevention in the post-LT setting, especially in combination with pharmacotherapy.^73^^,^^74^ Formalized studies of cognitive behavioral therapy, 12-step programs, and motivation-enhanced therapy demonstrated equal effectiveness in preventing alcohol use among individuals with AUD, although psychiatric comorbidity and lack of motivation remained challenges.^75^ Figure outlines a basic algorithm through which patients should be evaluated for LT and followed up with in a multidisciplinary manner. A comprehensive, protocolized follow-up model—one that includes structured relapse risk assessments, access to addiction treatment, frequent PEth testing, and regular multidisciplinary team input—has been associated with improved outcomes. For example, a study by Attilia et al. (2018) demonstrated that patients enrolled in a multidisciplinary support program that included structured post-LT monitoring, inpatient care for any “slip” events, and family involvement had significantly lower rates of relapse.^76^ Importantly, shorter duration of pre-LT program participation was associated with increased risk, underscoring the need for sustained engagement throughout the transplant continuum. Another Italian-based study showed that a multidisciplinary approach allowed for an earlier diagnosis of relapse and lower mortality.^77^

**Figure:**
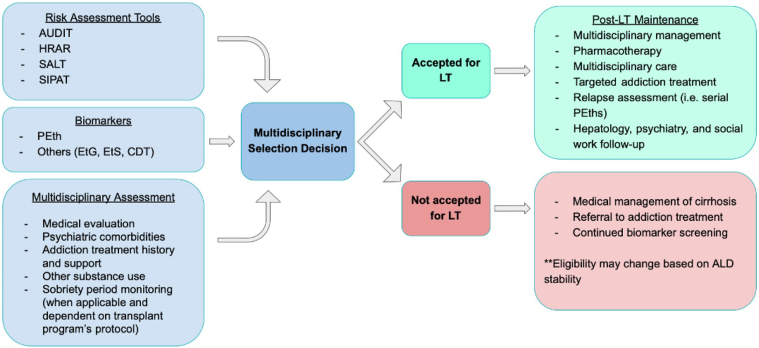
Algorithm for transplant selection: This figure illustrates key components of pretransplant evaluation—including structured risk assessment tools (AUDIT, HRAR, SALT, SIPAT), alcohol biomarkers (PEth, EtG, EtS, CDT), and comprehensive multidisciplinary assessment encompassing medical, psychiatric, and addiction-specific factors. These inputs inform the multidisciplinary selection decision, which results in acceptance or nonacceptance for LT. Patients approved for LT transition to structured post-transplant maintenance, including pharmacotherapy for alcohol use disorder, multidisciplinary follow-up (hepatology, psychiatry, and social work), and relapse monitoring (eg serial PEth testing). Patients not accepted for LT continue to receive medical management of cirrhosis, addiction treatment referral, and ongoing biomarker screening; eligibility may change depending on ALD stability. CDT, carbohydrate-deficient transferrin.

Despite these promising findings, significant gaps remain in the standardization and implementation of multidisciplinary care models. Variability across centers, lack of dedicated resources, and unclear guidelines regarding the frequency and content of psychosocial monitoring contribute to inconsistent patient experiences and outcomes. To address these challenges, there is a growing call in the literature for consensus-based protocols and further research into the components of effective post-LT relapse prevention programs.^10^

## Social Determinants of Health in ALD

Given the multifaceted nature of AUD, which intersects with mental health, socioeconomic conditions, and systemic inequities, understanding the influence of social determinants of health is essential when evaluating patients with ALD for LT. These individual and community-wide factors can significantly impact a patient’s ability to access care, meet transplant selection criteria, and engage in treatment post-transplant. Transplant programs, often under the pressure to demonstrate good outcomes, may unintentionally allow implicit bias or subjective judgments to influence decisions around candidacy and support.

### Education and Health Literacy

Educational attainment can influence health literacy, treatment engagement, and communication with medical providers. In the context of LT, health literacy may impact a patient’s understanding of their disease, recognition of the risks associated with ongoing alcohol use, and the importance of abstinence. One study found that patients without a high school diploma were more likely to be denied waitlisting during transplant evaluation.^78^ Similarly, individuals from counties with lower levels of educational attainment were less likely to be referred for transplant.^79^ These findings suggest that educational inequities may contribute to disparities in transplant access and highlight the need for equitable communication strategies during evaluation and beyond.

### Race and Ethnicity

While many chronic diseases disproportionately affect racial and ethnic minority groups, ALD presents a complex pattern. The impacts of race and ethnicity on care have long been studied, but not as much in the setting of LT, particularly in the context of relapse. Not only has access to LT been shown to be lower for ALD patients than it is for non-ALD end-stage liver disease patients in general, but this phenomenon disproportionately affects Black patients.^80^ This validates earlier findings demonstrating lower listing rates and greater numbers of deaths in Black patients compared to white patients.^81^

Interestingly, while countless medical conditions disproportionately impact minority groups, ALD seems to be unique in that post-LT relapse and AUD has been shown to be a growing concern in non-Hispanic White populations; a multicenter study of various post-LT populations found that the hazard ratio of shorter time to relapse for non-Hispanic Whites was 3.79 as compared to non-Whites, even after controlling for education, age, and psychiatric comorbidity.^9^ It is important to consider that despite these findings, there could be bias among selection committees and referrals of non-Hispanic White patients that may be inflating the degree to which this population has been shown to relapse.

### Sex

Sex, as a predictor of relapse itself, has not been shown to be an independent risk factor for post-LT relapse.^9^ However, gender disparities in the way research is conducted and how livers are allocated remain an ongoing concern. For example, almost all studies in ALD patients are predominantly male.^9^^,^^14^ Even some relapse risk scoring systems, such as the HRAR, were developed for use in, and therefore tailored to, the male veteran population.^23^

Intrinsically, metabolic and weight differences make females more susceptible to EtOH use, making them more likely to develop liver disease even with equivalent AUD severity and alcohol use as men.^82^ However, they remain underrepresented in the listing and transplant process.^80^ Even after LT, women with ALD also have worse 1-year survival than their male counterparts.^83^

### Socioeconomic Status and Geography

Although financial status is not a formal selection criterion for transplantation, socioeconomic position can impact a patient’s ability to access evaluation services, maintain abstinence, and demonstrate adequate psychosocial support. Several studies have explored the influence of broader structural metrics—including the Center for Disease Control and Prevention Social Vulnerability Index and the Social Deprivation Index—on transplant access.^84^ Patients residing in neighborhoods with high Social Deprivation Index scores or with higher rates of public insurance (Medicaid, Medicare, or Supplemental Nutrition Assistance Program participation) were significantly less likely to be listed for transplant and had lower transplant rates, even after adjustment for psychosocial risk using tools such as the SIPAT.^78^^,^^85^ In one study, counties with the lowest liver disease mortality had the highest rates of liver transplant referral, while those with the highest mortality had disproportionately lower referral rates and fewer gastrointestinal specialists.^79^ Insurance status was also found to be associated with referral likelihood, suggesting access gaps may be driven in part by healthcare coverage and provider availability.

Geographic variation further compounds these disparities. While the transplant community has long agreed that geography should not dictate access to organs, data suggest otherwise. In each United Network for Organ Sharing region, three transplant centers were found to account for up to 90% of that region’s transplants, creating a potential bottleneck in access depending on location.^30^ More recent work has used the AUD prevalence-to-transplant ratio and the alcohol-related liver disease death-to-transplant ratio as surrogate measures of LT accessibility. Both ratios demonstrated that regions with lower transplant center density have higher mortality relative to transplant rates.^86^^,^^87^ Similar trends have been reported internationally; for example, in the United Kingdom, greater distance to a transplant center is associated with worse outcomes.^88^

### Implicit Bias

The stigma associated with AUD—as a condition often perceived to result from personal choice—may introduce implicit bias into transplant decision-making. While transplant programs strive to apply objective criteria, concerns remain that subconscious judgments related to race, education, insurance status, or perceptions of “worthiness” may influence listing decisions for patients with ALD. Ongoing efforts to mitigate bias through standardized evaluation protocols, multidisciplinary review, and the use of objective biomarkers may help promote equity in transplant access and outcomes.

## Conclusion

Alcohol relapse after LT remains a critical challenge, but its risk cannot be reduced to a single factor or score. Instead, relapse emerges from the interaction of clinical history, mental health, psychosocial support, and broader structural conditions such as education, socioeconomic status, race, and geography. While existing scoring systems, such as SALT and SIPAT, provide some guidance, their limited accuracy and generalizability underscore the need to combine them with objective biomarkers like PEth and nuanced clinical judgment. Notably, the predictive validity of relapse models and post-transplant monitoring strategies has been even less studied in the living donor liver transplant setting, where patient selection dynamics and psychosocial environments may differ substantially from deceased donor transplantation. Beyond risk stratifications, the implementation of structured post-LT interventions, including addiction treatment, pharmacotherapy for AUD, and close multidisciplinary follow-up, has become critical to promoting long-term sobriety and optimizing outcomes. Importantly, the role of implicit bias and inequities in LT access must not be overlooked, as they can shape both candidacy decisions and outcomes. Moving forward, efforts to improve prediction and prevention of relapse should emphasize multidisciplinary care, standardized yet flexible assessment tools, and equity-driven approaches. By integrating biological, behavioral, and social perspectives, the field can better balance the imperative to safeguard scarce grafts with the responsibility to provide fair access to life-saving transplantation.
